# Sickness behaviour pushed too far – the basis of the syndrome seen in severe protozoal, bacterial and viral diseases and post-trauma

**DOI:** 10.1186/1475-2875-7-208

**Published:** 2008-10-14

**Authors:** Ian A Clark, Alison C Budd, Lisa M Alleva

**Affiliations:** 1 School of Biochemistry and Molecular Biology, Australian National University, Canberra, ACT 0200, Australia

## Abstract

Certain distinctive components of the severe systemic inflammatory syndrome are now well-recognized to be common to malaria, sepsis, viral infections, and post-trauma illness. While their connection with cytokines has been appreciated for some time, the constellation of changes that comprise the syndrome has simply been accepted as an empirical observation, with no theory to explain why they should coexist. New data on the effects of the main pro-inflammatory cytokines on the genetic control of sickness behaviour can be extended to provide a rationale for why this syndrome contains many of its accustomed components, such as reversible encephalopathy, gene silencing, dyserythropoiesis, seizures, coagulopathy, hypoalbuminaemia and hypertriglyceridaemia. It is thus proposed that the pattern of pathology that comprises much of the systemic inflammatory syndrome occurs when one of the usually advantageous roles of pro-inflammatory cytokines – generating sickness behaviour by moderately repressing genes (*Dbp, Tef, Hlf, Per1, Per2 *and *Per3*, and the nuclear receptor Rev-erbα) that control circadian rhythm – becomes excessive. Although reversible encephalopathy and gene silencing are severe events with potentially fatal consequences, they can be viewed as having survival advantages through lowering energy demand. In contrast, dyserythropoiesis, seizures, coagulopathy, hypoalbuminaemia and hypertriglyceridaemia may best be viewed as unfortunate consequences of extreme repression of these same genetic controls when the pro-inflammatory cytokines that cause sickness behaviour are produced excessively. As well as casting a new light on the previously unrationalized coexistence of these aspects of systemic inflammatory diseases, this concept is consistent with the case for a primary role for inflammatory cytokines in their pathogenesis across this range of diseases.

## Common ground in inflammatory disease, sickness behaviour and hibernation

The idea of acute infectious illness being caused by rampant overproduction of inflammatory cytokines that, in lower concentrations, mediate innate immunity, was first argued a quarter of a century ago [1], and has generated a large literature. Once recombinant tumour necrosis factor (TNF) and interleukin-1 (IL-1) became available in the late 1980s, and assays based on them replaced earlier methods, the concept spread to other pro-inflammatory cytokines, and from malaria and sepsis to viral and certain autoimmune diseases. It is now also well-entrenched in the literature of the post-trauma syndrome. Clearly, different triggers for cytokine generation and release can be expected to generate different foci, profiles, concentrations and kinetics of these mediators – now numerous enough to form superfamilies – and thus clinical variation within the same general principle is to be expected. But an accepted fundamental pattern has emerged.

At a time of shifting perceptions on the interaction between sickness and host activity (reviewed by Dantzer [2]), Hart [3] argued that the distinctive behaviour of sick humans and animals (lethargy, anorexia, depressed motor activity etc.) was not simply another untoward aspect of being ill. Instead, it was reasoned to be an adaptive syndrome that had evolved as a protective mechanism to maximize chances of survival through encouraging the sick animal to seek out and remain in a safe resting place, and not search for food, until a survivable infectious episode had passed. Hart also proposed that sickness behaviour was caused by the inflammatory cytokines, TNF and IL-1. This was later confirmed by others [4], and investigated further through showing that IL-1 contributes significantly to the anorexia caused by both endotoxin and influenza infection [5,6]. The literature on this field is now considerable. Indeed, Cavadini and co-workers [7] note that this link between TNF and IL-1 and sickness behaviour induced them to investigate if these inflammatory cytokines suppress expression of the clock genes that regulate circadian rhythm. It has recently [8] been realized that the circadian cycle controls many more biological functions than previously supposed.

Circadian rhythm and hibernation are variations of a theme of genetic control of activity and metabolism, and the degree to which these two clocks are functionally linked is often studied [9]. The first example of a human disease being thought of in these terms was ischaemic heart disease, in which the concepts were adopted to explain the self-protective downregulation of metabolic function seen in cardiomyocytes after repeated bouts of ischaemia. Like the seasonal hibernation seen in Arctic mammals, it involves biochemical changes directed at preserving energy, so in 1997 was termed myocardial hibernation [10]. Functional hibernation as a survival strategy in severe sepsis was proposed in 2003 [11], and subsequently documented in myocytes in the mouse caecal ligation and puncture model of systemic inflammatory disease [12]. Levy's recent reasoning [13] that broadens the possible relevance of cytokine-induced cardiomyocyte hibernation to other organs is, as Singer suggested [11], essentially sickness behaviour [3], with altered circadian rhythm being considered in terms of cellular bioenergetics. The consequence is to reduce demand for energy, in the form of ATP, to a level commensurate with its supply.

## Energy saving and the consequences of it being overdone

As outlined above, it seems reasonable that sickness behaviour, including lowering the metabolic rate to conserve energy and turning off appetite to avoid the urge to forage, will help tide the ill animal or human over an illness crisis. Recently, Cavadini and co-workers [7] reported that TNF and IL-1β suppress the expression of genes (*Dbp, Tef, Hlf, Per1, Per2 *and *Per3*) *in vivo*, and a nuclear receptor (Rev-erbα) involved in controlling circadian rhythm. The authors proposed that these cytokines thereby provide a link between the fatigue associated with autoimmune diseases, such as rheumatoid arthritis and Crohn's disease.

Nevertheless these cytokines, and others related to them, can cause a much wider spectrum of harmful changes than this [14]. Why these changes, so familiar from the literature and everyday observation, should occur as a group, forming a distinct syndrome, currently lacks a rationale. It was therefore considered how widely the implications of severe sickness behaviour, through suppressed circadian rhythm [7], might extend, and whether this approach might be able to explain the composition of the severe systemic inflammatory syndrome common to malaria, sepsis, viral infections, and also seen post-trauma. The literature to date has focussed on mechanisms for individual changes, but not questioned why the mosaic contains the components one is accustomed to observing.

**Figure 1:**
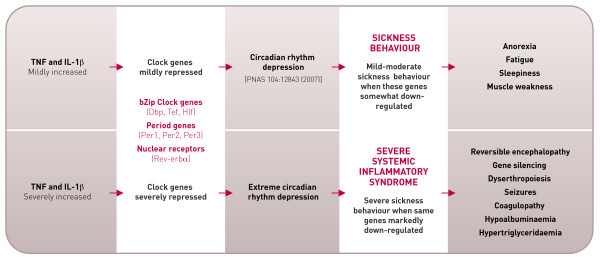
**The disease consequences of extreme circadian rhythm gene repression**. A comparison of the mild and severe consequences of *in vivo *repression of PAR bZip clock genes (*Dbp*, *Tef *and *Hlf*), period genes (*Per1*, *Per2*, and *Per3*) and a nuclear factor (Rev-erbα) by inflammatory cytokines.

In other words, are inflammatory cytokines simply innately harmful in excess, pathophysiology arising from excessive manifestation of the physiological roles they possess when in trace amounts? Or, as suggested here (Figure 1), do the wider functions of the nuclear receptor (Rev-erbα) and genes (*Dbp, Tef, Hlf, Per1, Per2 *and *Per3*) [7] lead to an understanding of the syndrome to be expected when a severe inflammatory illness occurs? In documenting this suppression Cavadini and co-authors administered, to mice, only a tenth of the dose of bacterial lipopolysaccharide (LPS) required for a severe response in this species. Accordingly, the literature was examined to see whether these cytokine-suppressed nuclear receptor and genes involved in circadian rhythm have other functions that help us understand the pattern of changes routinely observed in the systemic inflammatory syndrome.

## Reversible encephalopathy

An active brain consumes much more energy than any other organ of similar size, largely through the demands of the Na^+^/K^+ ^ATPase that runs the cell membrane Na^+^/K^+ ^pump [15]. Hence, it is likely to have been a prime conservation site for any set of adaptive changes that have evolved to conserve energy in order for the organism to survive, whether at the level of sickness behaviour or hibernation, both of which can be conveniently approached in terms of altered circadian rhythm. The fatigue of chronic inflammatory diseases such as rheumatoid arthritis is consistent with the capacity of TNF, to which this fatigue is linked in the literature, to suppress circadian rhythm genes [7]. Sleep saves energy and pathways that lead to sleep are induced by TNF [16] or IL-1β [17]. Also, electroencephalogram (EEG) delta wave power is decreased for several days when short interfering RNA (siRNA) targeting TNF is microinjected into the primary somatosensory cortex [18]. This is consistent with TNF increasing cortical EEG delta wave power and, therefore, [19] being involved in sleep regulation.

The TNF and IL-1β-susceptible genes through which these changes are controlled have not been identified, but the above observations imply the same principles apply as the need to reduce ATP expenditure increases in urgency with more severe illness, progressing through the increased fatigue and sleep periods to the clinical signs of encephalopathy, including coma, in severe malaria, sepsis, or influenza. Clearly, this encephalopathy can be associated with a harmful outcome in these systemic inflammatory states, but its evolutionary function may simply be to save energy. Indeed, this strategy is likely to be successful in, for example, most adult cerebral malaria patients, who recover with negligible neurological deficit if other organ failure is not involved [20]. TNF [21] and IL-1 [22] are increased in proportion to the degree of coma in falciparum malaria. The attractiveness of impaired consciousness in this disease being based on awareness of the basic physiology and broad effects of these and functionally similar cytokines across acute disease in general – rather than on primary vascular blockage by sequestered parasitized red cells [23] – is enhanced by recent evidence, from Papua New Guinea [24] and West Papua [25], of the essentially non-sequestering *Plasmodium vivax *causing this encephalopathy to the same degree as does *Plasmodium falciparum*.

How might this coma come about? Biology's known gaseous signalling molecules offer possibilities, since they are increased in inflammatory states, and induce a hibernation-like reversible state of suspended animation that protects against low ATP levels in *Drosophila melanogaster *larvae (NO [26]), *Caenorhabditis elegans *(CO [27]) and mice (H_2_S [28,29]). All three gases have the potential to operate through inhibiting mitochondrial cytochrome oxidase [30-32]. A striking contrast exists, however, in the roles of these three gases in mammalian inflammation – both NO [33] and CO [34] inhibit NF-κB, whereas H_2_S upregulates it [35]. This dichotomy may prove to explain why both NO and CO are anti-inflammatory in mice, including protecting against the mouse model of malaria encephalopathy [36,37], whereas H_2_S is pro-inflammatory, increasing plasma TNF levels in mice [38], and its inhibition protects in sepsis models [38,39]. These three gases warrant a detailed comparative investigation in this context.

## LPS tolerance and inflammation-induced gene silencing

A major unaddressed question is the origin of the widespread gene silencing that develops after the initial illness crisis of severe systemic inflammation [40], including H5N1 influenza in mice [41]. Immunosuppression is common to haemoprotozoan diseases such as malaria [42] and trypanosomiasis [43], as well as in sepsis [44], influenza [45], and trauma [46]. Patients who survive the initial acute effects of excessive levels of inflammatory cytokines often succumb during a subsequent period of immunological and metabolic shutdown, in a state variously termed anergy, immune paralysis, or monocyte deactivation, with monocytes unable to generate HLA antigens or enough cytokines for normal immunological responses [47], let alone the excess that might be harmful to the host [48]. In short, a systemic hyperinflammatory response turns into a harmful hypoinflammatory state of unknown origin [49]. Explanations for these events in sepsis have included anti-inflammatory cytokines out-producing inflammatory cytokines [50] and severe lymphoid cell apoptosis [51]. However, both pro- and anti-inflammatory cytokines proved to switch off as the disease progresses [52], and the point of maximum T cell anergy correlates to a diminished apoptotic response [53].

LPS tolerance is a common model for gene silencing, and repressed gene expression, arising from disrupted transcription, is as widespread in LPS tolerance as in severe inflammatory disease. Indeed the two coincide, with patients recovering from malaria being tolerant to LPS [54] as well as to high loads of malaria parasites [55]. In landmark studies in 1975 Lewis Thomas widened our awareness of the broad relevance of the changes seen in LPS tolerance by studying it in parallel to tolerance to haemorrhagic shock and trauma shock [56]. Arguably, therefore, LPS tolerance is simply a particular way of detecting a process common to many disease states. For instance, the patterns of NF-κB expression in sepsis and LPS tolerance closely resemble each other [57], and monocyte inactivation is as generalized, with the same characteristics, in LPS tolerance as in chronic sepsis [58]. LPS tolerance, and therefore sepsis gene silencing, has a functional link to the theme of this review, in that it has a distinct circadian rhythm [59,60], and can be created by depleting mice of *Per2*, one of the circadian genes that TNF suppresses [7].

More recently, LPS tolerance has been explained in terms of RelB induction generating transcriptionally inactive NF-κB p65/RelB heterodimers [61] and epigenetic silencing of TNF [62]. The latter seems a particularly cogent argument, with LPS tolerant, or silenced, cells exhibiting repressed production of TNF mRNA, retained binding of heterochromatin binding protein 1α, sustained methylation of histone H3 lysine 9, reduced phosphorylation of histone H3 serine 10, and diminished binding of NF-κB RelA/p65 to the TNF promoter. This group has combined these elements, which are consistent with basic studies on gene silencing [63], into a compelling case to explain this phenomenon in severe systemic inflammation [40]. It is, moreover, consistent with the hypoinflammatory state being a logical consequence of the earlier cytokine storm that caused the excessive sickness behaviour.

## Changes less likely to have survival advantage

### Dyserythropoiesis

A significant contribution is made to anaemia, in both acute and chronic inflammatory states, by defective generation of new red cells in bone marrow, or dyserythropoiesis. It can be reproduced by injecting TNF [64], and not surprisingly has been recorded in a range of infectious states in which pro-inflammatory cytokines are increased, including malaria [65], trypanosomiasis [66] and viral diseases [67-69]. As in many other tissues, the bone marrow's haemopoietic cells are governed by time-dependent variations in clock gene expression. This includes Per1, Per2, and the nuclear receptor Rev-erbα [70], all three of which are included in the nuclear elements shown to be suppressed by TNF [7]. This suppression gives a pathway whereby TNF could reduce haemopoietic activity in infectious diseases. It can be viewed as part of a generic attempt to save energy, in which any initial savings would soon become a liability as haematocrit, and therefore the capacity to carry oxygen in the circulation, falls.

### Seizures

Febrile seizures are traditionally attributed to fever itself, rather than fever and seizures being visualized as having a common cause. Seizures are particularly common in malarial illness. In one large study [71] based in Kenya, 69% of patients experienced them, and other reports from West Africa record a considerably higher incidence. They are also frequently observed in paediatric sepsis [72], burn injury [73], and a range of acute severe viral diseases, including influenza [74] and Lassa fever [75].

An unexpected development in understanding the origin of certain types of seizures came from the realization, several years ago [76], that mice with deletions of the genes for three circadian PARbZip transcription factors, DBP, HLF and TEF, are highly susceptible to generalized spontaneous and audiogenic seizures. Moreover, fits were four times as likely to occur in the major sleep period of the circadian cycle than in its major active period. These authors identified *Pdxk *as a target gene of these transcription factors, noting the role of this gene in the generation of pyridoxal phosphate, a coenzyme in the synthesis of neurotransmitters such as γ-aminobutyric acid, serotonin and dopamine. Through the genes for these three transcription factors being among those recently reported to be suppressed by TNF and IL-1β [7], this mechanism for seizures is now linked to infectious disease and fever seizures, since these and similar pyrogenic cytokines dominate disease pathogenesis. These authors found an appreciable degree of down-regulation by a dose of cytokine a tenth of that required to make mice seriously ill, so these two cytokines warrant investigation as the cause of seizures, through *Dbp, Tef, and Hlf *downregulation, in circumstances in which levels of these cytokines are raised, such as severe haemoprotozoal, bacterial and viral diseases.

### Coagulopathy

Coagulopathy is another characteristic component of the systemic inflammatory syndrome. As well as being detectable in human volunteers receiving parenteral TNF [77], it is, like seizures, common to malaria [78], sepsis [79], and viral diseases (eg dengue [80], Ebola [81] and influenza [82]). Plasminogen activator inhibitor type 1 (PAI-1), which retards the generation of plasmin, thereby adding to coagulopathy by slowing clot dissolution, is a major regulator of the fibrinolytic system. It is expressed with a circadian rhythmicity, peaking in the early morning. The nuclear receptor Rev-erbα, a core component of the circadian loop, causes this cyclic expression of human PAI-1 gene expression through two Rev-erbα binding sites in the PAI-1 promoter [83]. Hence, the capacity of TNF to suppress Rev-erbα, and thus circadian rhythm [7], may also explain why it enhances coagulopathy [77], and why this occurs in protozoal, bacterial, and viral diseases. Higher levels of PAI-1 predict a poor outcome in patients with sepsis [84]. Thus, it is argued here that the coagulopathy of these systemic disease states is a further example of sickness behaviour, i.e. circadian rhythm shutdown, taken to harmful extremes.

### Hypoalbuminaemia

Plasma albumin has several important biological functions, including being an extracellular transition metal ion-binding and radical-scavenging antioxidant, and an important contributor to plasma osmolarity. Its level is therefore normally controlled within tight limits. Levels are characteristically low in systemic inflammatory states, and this decrease can also be accounted for by this proposal. Hypoalbuminaemia occurs routinely in malaria [85], sepsis [86,87], various severe viral diseases (eg Korean haemorrhagic fever [88], dengue [89], Ebola [90], viral hepatitis [91], SARS [92]), visceral leishmaniasis [93] and trauma [94]. Serum albumin level is an independent indicator of outcome in severe sepsis [95], so much so that it was included in the criteria when the APACHE scoring system was upgraded from II to III [96]. The liver-specific albumin gene is positively regulated by *Dbp *[97], one of the circadian genes that TNF suppresses [7]. TNF has been demonstrated, in picomolar concentrations, to reduce albumin production by human hepatocytes [98].

### Hypertriglyceridaemia

Usually, and for sound metabolic reasons, the concentration of triglycerides in plasma is closely regulated. Circulating levels can be increased by exogenous TNF [99], and this cytokine has been shown to bring this about in thermal injury [100]. Thus hypertriglyceridaemia can be expected in diseases in which TNF is acutely increased, as documented in malaria [101] and sepsis [86,102]. However, no reference exists to circulating triglyceride levels having been examined in viral diseases. Since TNF suppresses the nuclear receptor Rev-erbα [7], and hypertriglyceridaemia occurs in Rev-erbα KO mice [103], the presence of this change in the systemic inflammatory syndrome is predictable.

## Consistent with a primary cytokine origin of systemic disease

These arguments strengthen the concept that the pro-inflammatory cytokines are the primary driver of the pathophysiology of severe infectious, including malaria, and post-trauma illness. An alternative view, that each pathogen causes disease through a different primary mechanism (eg death of viral-infected host cells or vasculature blockage by parasitized red cells in malaria [23]), with cytokines providing only non-specific changes such as fever, still exists. This view is further weakened by the above literature and reasoning, since it is difficult to credit vascular obstruction (falciparum malaria) or cell death (viral infection) with the ability to generate the above changes, such as coagulopathy, hypoalbuminaemia or immunosuppression, in a single infectious disease, let alone a range of them, or post-trauma.

## Conclusion

While sickness behaviour, comprising a series of changes caused through pro-inflammatory cytokines shutting down circadian rhythm, has survival advantages, its more intense expression in severe disease generates a range of more harmful alterations, recognizable as the severe systemic inflammatory response. These are controlled by more severe effects of the same cytokines (TNF and IL-1β) affecting the same genes and nuclear receptor(s) as suppress circadian rhythm. Accordingly, the coexistence of reversible encephalopathy, gene silencing, dyserythropoiesis, seizures, coagulopathy, hypoalbuminaemia and hypertriglyceridaemia in severe infectious disease, including malaria, can best be understood as an extreme form of sickness behaviour. It is further proposed that this pattern, familiar in such conditions, has persisted in evolution because it has a survival advantage when, most commonly, the syndrome is less severe. In terms of a basis for developing treatments for human disease, this implies that the observed hypoinflammatory changes seen in these conditions require no primary explanation other than they are logical consequences of excess earlier production of pro-inflammatory cytokines, although local variations in secondary, cytokine-induced mechanisms occur.

As knowledge of the range of genes controlled by the superfamilies of pro-inflammatory cytokines and the genetic control of pathological changes both expand, it seems likely that other components of the systemic inflammatory syndrome will be added to the list begun in this paper.

## Competing interest

The authors declare that they have no competing interests.

## Authors' contributions

All three authors contributed to wide-ranging discussions on the ideas contained in this manuscript. IC wrote the manuscript, to which LA and AB made invaluable suggestions.
